# Exploring the Association between Elevated Anxiety Symptoms and Low Skeletal Muscle Mass among Asymptomatic Adults: A Population-Based Study in Republic of Korea

**DOI:** 10.3390/brainsci14050438

**Published:** 2024-04-28

**Authors:** Eunsoo Kim, Sra Jung, Mi Yeon Lee, Chul-Hyun Park, Sung Joon Cho

**Affiliations:** 1 Department of Psychiatry, Kangbuk Samsung Hospital, Sungkyunkwan University School of Medicine, Seoul 03181, Republic of Korea; silverstream421@gmail.com; 2 Department of Psychiatry, Cha University Ilsan Medical Center, Goyang 10223, Republic of Korea; srajung890@chamc.co.kr; 3 Division of Biostatistics, Department of Academic Research, Kangbuk Samsung Hospital, Sungkyunkwan University School of Medicine, Seoul 03181, Republic of Korea; my7713.lee@samsung.com; 4 Department of Physical and Rehabilitation Medicine, Kangbuk Samsung Hospital, Sungkyunkwan University School of Medicine, Seoul 03181, Republic of Korea; 5 Workplace Mental Health Institute, Kangbuk Samsung Hospital, Seoul 03181, Republic of Korea

**Keywords:** anxiety symptoms, low skeletal muscle mass, mental health, sarcopenia, aging

## Abstract

Individuals with mental health problems are at higher risk of musculoskeletal diseases. However, the association between low muscle mass (LMM) and anxiety symptoms remains uninvestigated. This cross-sectional study enrolled 174,262 adults (73,833 women, 100,429 men), aged 18 to 89, who completed the anxiety scale and body composition analyses. Using bio-electrical impedance analysis, skeletal muscle mass index (SMI) was calculated based on appendicular skeletal muscle mass (ASM) (kg)/height (m^2^). LMM was defined as SMI < 7.0 kg/m^2^ in men and <5.4 kg/m^2^ in women. Anxiety symptoms were screened using the Clinical Useful Anxiety Outcome Scale (CUXOS) with cut-off scores of 20, 30, and 40. Multivariable logistic regression analyses were performed. LMM prevalence was 20.17% in women, 3.86% in men (*p* < 0.001). The prevalence of anxiety symptoms in LMM group decreased from mild (CUXOS > 20: women, 32.74%, men, 21.17%) to moderate (CUXOS > 30: 13.34%, 7.32%), to severe anxiety symptoms (CUXOS > 40: 4.00%, 1.73%). In multivariable-adjusted models, LMM was associated with mild (aOR (95% confidence interval)), women, 1.13 (1.08–1.17); men, 1.17 (1.08–1.27)), moderate (1.17 (1.11–1.24); 1.35 (1.19–1.53) and severe anxiety symptoms (1.18 (1.07–1.3), 1.36 (1.06–1.74)), demonstrating an increased risk of ORs with escalating anxiety severity. LMM was independently associated with a higher prevalence of anxiety symptoms.

## 1. Introduction

In recent decades, “aging”, with dramatic increases in human life expectancy, has become a natural trend in modern society. Among the aging processes, musculoskeletal changes, such as decreases in bone and muscle mass and increases in fat mass, are identified as a major contributor that hinders autonomy and affects the overall quality of life in the future. Sarcopenia, etymologically meaning “poverty of flesh” [1], is defined by low levels of measures for three parameters: (1) muscle strength, (2) muscle quantity/quality and (3) physical performance as an indicator of severity [2]. A recent large meta-analysis study from the United Kingdom found that the overall average was 8–36% for those under the age of 60 years and 10–27% for those over 60 years of age [3]. While sarcopenia has traditionally been thought to be a consequence of aging, recent studies have shown that it is not uncommon in younger individuals because of malnutrition [4], infection, anorexia nervosa [5], diabetes [6] and malignancies [4,6,7]. Sarcopenia in all age groups is associated with more falls and fractures; trouble managing chronic diseases such as cardiovascular diseases, and stroke [6,8];and poor mental health outcomes such as degenerative cognitive disorders [9,10,11,12] or depression [13,14,15,16], resulting in loss of independence, diminished quality of life, hospitalization and mortality [17,18]. Skeletal muscle mass loss mainly attributes to the reduction in contractile force of muscle cells (single myofibrils), rather than qualitative decline, such as changes in myofiber morphology [19]. Low skeletal muscle mass (LMM) is a poor prognostic factor when accompanied by various diseases such as malignancies, secondary pulmonary fibrosis, coronary artery disease and abdominal surgery [20,21,22,23,24,25]. Even as a single factor, the presence of LMM requires careful clinical management.

Anxiety disorders are biopsychosocial conditions distinguished by disproportionate and enduring fear of everyday occurrences alongside behavioral and somatic symptoms [26]. Anxiety disorders are the most common psychiatric disorders worldwide [27], with lifetime prevalence rates ranging from 14.5% to 33.7%, according to a large prevalence study conducted in the United States [28]. Anxiety disorders vary in clinical presentation, with some individuals developing phobias triggered by specific environmental factors, while others experience severe episodic distress, as seen in panic disorder [29]. Anxiety disorders typically persist chronically, leading to increased healthcare utilization, and elevated rates of sick leave and retirement [30]. Coupled with their high prevalence rates, these factors result in a significant societal and economic burden.

The link between anxiety disorders and chronic physical illness is well documented around the world, with similar findings from the National Comorbidity Survey (NCS) in the United States, Canada, the Netherlands, Germany and the United Kingdom [31,32,33,34]. Few previous studies suggested that anxiety disorder is associated with sarcopenia [30,31] However, the consistency of the relationship between anxiety symptoms and sarcopenia remains inconclusive [35,36,37]. Several cohort and cross-sectional studies have reported an association between poor muscle strength and the risk of generalized anxiety disorder [38,39,40]. However, these studies have been limited to muscle function, making it difficult to confirm an association for muscle mass. The relationship between muscle mass and anxiety symptoms can only be found within specific disease groups such as hemodialysis patients [41] and cancer patients [4,7]. In addition, previous studies have shown a strong association between sex and loss of muscle mass [42] and demonstrated that sex hormones play an important role in muscle metabolism [3], including changes in sugar metabolism, fat oxidation and inflammation levels [3,43,44]. 

Given the high prevalence of anxiety disorders in the general population and the common coexistence of anxiety disorders and chronic physical illness, it is worthwhile to identify the association between anxiety symptoms and skeletal muscle mass loss. As the muscle mass differs between men and women, we further hypothesized that there were sex-specific differences in anxiety patterns that have not been investigated to date. Therefore, this study aims to analyze the association between LMM and anxiety symptoms in a healthy general population and to determine whether there are sex-differences in the association between LMM and anxiety symptoms.

## 2. Materials and Methods

**Figure 1:**
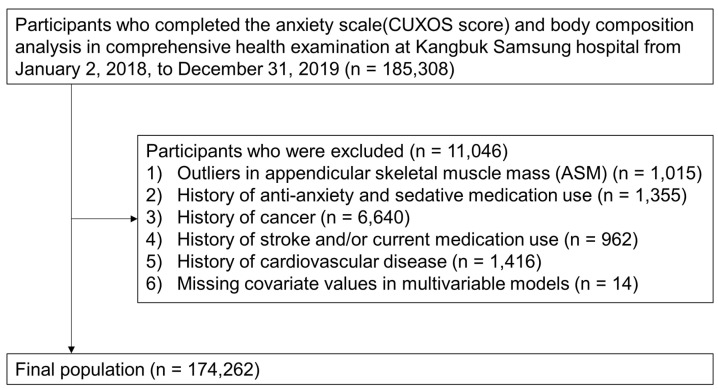
Selection of study population.

This cross-sectional study was conducted in two Kangbuk Samgsung Hospital Screening Centers in Seoul and Suwon. A total of 185,308 participants (aged 18–89 years) who completed the body composition analysis were recruited from primary healthcare screening programs between 2 January 2018 and 31 December 2019. After excluding 11,046 participants who met one or more of the exclusion criteria, 174,262 participants (73,833 women and 100,429 men) were analyzed to determine the association between LMM and anxiety symptoms. The exclusion criteria included: (1) outliers in appendicular skeletal muscle mass (ASM) (*n* = 1015), (2) history of anti-anxiety and sedative medication use (*n* = 1355), (3) history of cancer (*n* = 6640), (4) history of stroke and/or current medication use (*n* = 962), (5) history of cardiovascular disease (*n* = 1416) and/or (6) missing covariate values in multivariable models (*n* = 14, fasting plasma glucose = 1, systolic blood pressure = 12, high-density lipoprotein cholesterol (HDL-C) = 2) (Figure 1). Finally, the study protocol was approved by our hospital’s Institutional Review Board (IRB) (IRB No. KBSMC 2022-07-017). An IRB waiver from the requirement for informed consent was obtained as de-identified samples routinely collected as part of a health screening test were used.

### 2.1. Measurements

This study used the self-administered Korean version of the Clinically Useful Anxiety Outcome Scale (CUXOS) to measure the severity of anxiety symptoms. Previous studies have reported an internal consistency of 0.90 and test–retest reliability of 0.74 for the Korean version of the CUXOS. The questionnaire consists of 20 items, including 6 items in the mental anxiety domain and 14 in the physical anxiety domain, and participants respond to each item using a 5-point Likert scale. Each item was scored 1–4, with an overall score of 0–80, and the level of anxiety (CUXOS < 10 no anxiety; 11–20 minimal anxiety; 21–30 mild anxiety; 31–40 moderate anxiety; >40 severe anxiety) was defined based on previous research [45,46].

ASM (kg) was measured as the sum of arm and leg muscle mass using bio-electrical impedance analysis (BIA, In Body 720, Biospace, Seoul, Republic of Korea). A previous study reported that BIA predicts valid estimates of skeletal muscle mass in adults [47]. The BIA was calibrated each morning before testing and validated for reproducibility and accuracy for skeletal muscle mass analysis. Skeletal muscle mass index (SMI) was calculated using the following formula: SMI (kg/m^2^) = ASM (kg)/height (m)^2^ (41). Low skeletal muscle mass (LMM) was defined according to the Asian Working Group for Sarcopenia (AWGS) criteria (<5.7 kg/m^2^ for women and <7.0 kg/m^2^ for men) [48].

Demographic characteristics; health-related behavioral variables (smoking history, alcohol consumption, physical activity); and medical history (history of cancer, cardiovascular disease, stroke, hypertension, diabetes, and hyperlipidemia) were collected by examining physicians using a self-administered standardized questionnaire. Participants with history of anti-anxiety and sedative medication use were recruited based on their affirmative response to the question, “Have you ever taken anti-anxiety or sedative medication for a week or more within the past month?”. Participants with a history of smoking were categorized as never smokers, former smokers or current smokers. Current smokers were individuals who were actively smoking during the health examination and had smoked over 100 cigarettes in their lifetime. Former smokers were defined as individuals who had smoked over 100 cigarettes in their lifetime but were not currently smoking at the time of the interview. Participants who did not meet both criteria were classified as non-smokers [26]. Participants with an alcohol consumption of 20 g or more per day were categorized as heavy drinkers [49]. Physical activity was assessed using the International Physical Activity Questionnaire-Short Form (IPAQ-SF). Participants who engaged in vigorous exercise at least three times per week for at least 20 min per session were categorized as engaging in regular physical activity following the 2020 WHO guideline [50]. Trained nurses or medical laboratory technicians performed blood samples and anthropometric measurements. The lipid profile was measured by an enzymatic colorimetric assay, and blood glucose was measured by the hexokinetic method. Triglycerides were measured using an enzymatic calorimetric test, and total cholesterol levels were determined. According to the Hypertension Detection and Tracking Program protocol, blood pressure was measured systolic and diastolic using a standard sphygmomanometer after 5 min of rest. A history of hypertension was defined as a blood pressure of 140/90 mm Hg or higher or current use of antihypertensive medication according to the criteria of the Eighth Report of the Joint Committee on Prevention, Detection, Evaluation, and Treatment of High Blood Pressure. Body mass index (BMI) was estimated as weight in kilograms divided by meters squared (kg/m^2^).

### 2.2. Statistical Analysis

The baseline characteristics of the study population were analyzed using multiple-variable linear regression for continuous variables and the χ^2^ test for categorical variables. The prevalence of LMM according to the presence and severity of clinical anxiety symptoms assessed by CUXOS was compared using the χ^2^ test. Multiple logistic regression analysis was performed to determine the association of LMM with mild anxiety (CUXOS > 20), moderate anxiety (CUXOS > 30) and severe anxiety (CUXOS > 40). Three models adjusted for confounding factors were used. The adjustment for each model was as follows: model 1, adjusted for age, and screening center; model 2, adjusted for model 1 plus hypertension, fasting plasma glucose; and model 3, adjusted for model 2 plus smoking status, heavy alcohol consumption and regular physical activity. The mean CUXOS values of the control and LMM groups were compared using an analysis of covariance (ANCOVA) after adjusting for confounding variables. Statistical significance was set at a two-tailed probability of significance (*p*-value < 0.05) [51]. All analyses were performed using STATA version 17.0 (IBM Co., Armonk, NY, USA). 

## 3. Results

### 3.1. Baseline Characteristics

A total of 174,262 participants were included, comprising 73,833 women and 100,429 men. In women and men, the mean age was 41.57 (SD, 8.42) and 42.75 (8.5) years, respectively (*p* < 0.001), and the mean SMI was 6.2 (0.61) and 8.1 (0.65) kg/m^2^ (*p* < 0.001) (Table 1). When classifying the control and LMM groups according to gender, 14,891 (20.17%) of the participants were in the LMM group, and 3878 (3.86%) were in the control group. All variables were significantly different between the control and LMM groups in both women and men (*p* < 0.005), except for the total cholesterol level in women (Table 2).

### 3.2. Association between Severity of Low Skeletal Muscle Mass and Anxiety Symptoms (CUXOS Score)

Among the total participants, 41,680 (23.92%) were found to have mild anxiety symptoms, and the prevalence of mild anxiety symptoms was higher among women (22,659, 30.69%) compared to men (19,021, 18.94%). The prevalence of anxiety symptoms in LMM group decreased as the severity of anxiety symptoms increased from mild anxiety symptoms (women, 32.74%, men, 21.17%) to moderate anxiety symptoms (women 13.34%, men, 7.32%), to severe anxiety symptoms (women, 4.00%, men, 1.73%) (Supplementary Table S1).

Table 3 shows the univariate and multivariable logistic regression analyses for the association between LMM and anxiety symptoms. When the univariate logistic analysis was performed, LMM was highly associated with mild anxiety (women, adjusted odd ratios (aOR) 1.13, 95% CI 1.08–1.17; men, aOR 1.16, 95% CI 1.07–1.25); moderate anxiety (women, aOR 1.18, 95% CI 1.12–1.24; men, aOR 1.29, 95% CI 1.14–1.46); and severe anxiety (women, aOR 1.2, 95% CI 1.09–1.32; men, aOR 1.27, 95% CI 0.99–1.63). After adjustments for possible confounding factors, including age, screening center in Model 1, hypertension and fasting plasma glucose in Model 2, the aOR was generally fluctuated, but statistical significance was still observed in most values. When smoking status, heavy alcohol consumption and regular physical activity were added to Model 2 to identify the effects of health-related behavior factors, a similar statistical significance was observed in Model 3. LMM was associated with mild anxiety (women, aOR 1.13, 95% CI 1.08–1.17; men, aOR 1.17, 95% CI 1.08–1.27); moderate anxiety (women, aOR 1.17, 95% CI 1.11–1.24; men, aOR 1.35, 95% CI 1.19–1.53); and severe anxiety (women, aOR 1.18, 95% CI 1.07–1.3; men, aOR 1.36, 95% CI 1.06–1.74), independently (Table 3). In both women and men, there was an increased risk of aORs from mild to moderate to severe anxiety symptoms. The association between LMM and anxiety symptoms was significant in all models, with the highest association in Model 3 and the association in men was generally stronger than in women.

### 3.3. Comparison of Anxiety Level between the Control Group and Sarcopenia Group

**Figure 2:**
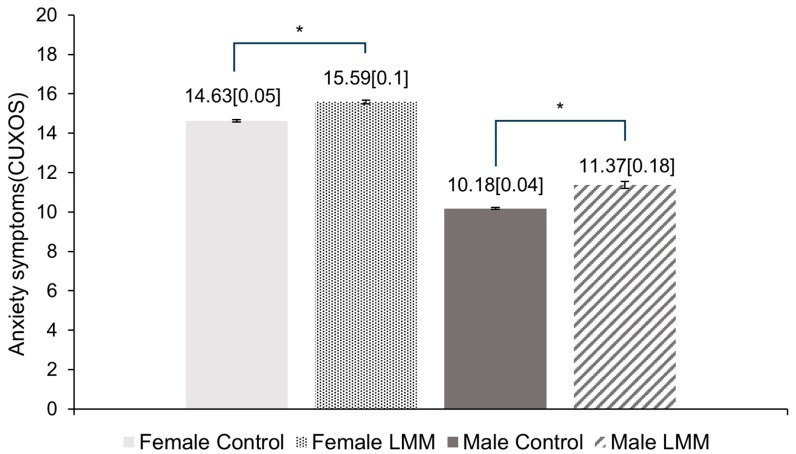
Comparison of adjusted mean of CUXOS score between Control and LMM groups in Model 3. Adjusted means (SE) of natural-log-transformed CUXOS scores in the groups were estimated from ANCOVA after adjustments for age, center, history of hypertension, fasting plasma glucose, smoking status, heavy alcohol and regular physical exercise. *: Group difference by independent *t*-test *p* < 0.001. LMM: low skeletal muscular mass; SE: standard error.

Figure 2 compares CUXOS values with and without LMM for women and men in Model 3. The CUXOS values in women was higher than that in men. In women, after adjusting for all confounding variables such as age, center, hypertension, diabetes, smoking, alcohol and regular physical activity, CUXOS values were observed to be significantly higher in the LMM group (15.59 (0.1), adjusted mean (standard error)) than in the control group (14.63 (0.05)). (Adjusted *p*-value < 0.001.) Similarly, for males, a significantly higher CUXOS value was observed for the LMM group (11.37 (0.18)) than the control group (10.18 (0.04)). (Adjusted *p*-value < 0.001.)

## 4. Discussion

To the best of our knowledge, this is the first study to examine the association between low skeletal muscle mass (LMM) and anxiety symptoms in a healthy general population. This study identified a significantly increased risk of anxiety symptoms with LMM, even after adjusting for multiple confounding variables. Interestingly, while the level of anxiety symptoms was higher in women, the association between LMM and anxiety symptoms was generally stronger in men.

In the Irish longitudinal study on ageing (TILDA) study, a long-term cohort aging study of local residents aged 50 years and older in Ireland, low hand strength suggestive of sarcopenia (<30 kg for men and <20 kg for women) was associated with an increased risk of depression (OR = 1.44; 95% CI: 1.08–1.92), as well as anxiety symptoms (OR = 1.61; 95% CI: 1.20–2.14) [38]. A 1-SD increase in strength was associated with 12.1% lower odds of GAD coexistence [39]. Previous studies have confirmed the association of increased muscle strength with mental illnesses, such as cognitive decline [10,11,12] and depression [10,13,15,16]. However, despite LMM being primarily implicated in the reduction of muscle contractility [19] and associated with adverse outcomes in chronic diseases [20,21,22,23,24,25], there is no direct evidence confirming its association with anxiety symptoms. Another drawback of the previous studies is the exclusive focus on older adults, which limits their generalizability. Considering the decline of 3–8% in muscle mass every decade after the age of 30 [52], alongside the observation of decreased muscle activity, ongoing physiological and pathological processes, and systemic inflammation in young individuals [53,54], this study holds value in that it encompasses all age groups. 

Our findings demonstrated that LMM was associated with anxiety symptoms, and this association strengthened as the severity of anxiety symptoms increased. The first possibility for this association between LMM and elevated anxiety symptoms is that people with LMM experience functional limitations and impairments that make it difficult for them to carry out their daily activities [18], and therefore experience reduced activity levels, social isolation, low self-esteem and poor quality of life [17], which can lead to increased anxiety and depressive symptoms [55,56]. A study using data from the National Survey of Mental Health and Well-being (NSMHWB), a national survey that assesses mental illness and chronic physical illness in Australian adults, found that 32.2% of 8841 Australian adults had a chronic medical condition, with 21.9% having a comorbid anxiety disorder. Mood and anxiety disorders were 1.5 and 1.8 times more common in the group with chronic physical conditions, respectively (affective OR 1.5; anxiety OR 1.8) [6]. Previous studies of the association between musculoskeletal disorders and mental illness have shown that mood disorders are associated with low bone mineral content and anxiety disorder is associated with cervical or lumbar disc disease [32]. One study that looked at hemodialysis patients and linked LMM to poor nutrition, anxiety and depression found that LMM was associated with higher levels of depression and anxiety. In comparison, higher muscle mass was associated with better physical function and general health [31]. Another possibility is based on pathophysiological hypotheses associated with neuroactive substances. When muscles contract, cytokines and myokines are released into the circulation, and the hypothesis is that these neurotransmitters play a role in preventing depression and anxiety disorder [57]. Neurotrophins (NFs) released by contracting skeletal muscle maintain the size and number of skeletal muscles while providing nutrients for nervous system growth and differentiation, which may help with depression and anxiety disorders. Furthermore, skeletal muscle activity has immune and redox effects that reduce muscle catabolism [35,58]. In line with these studies, positive psychological states are associated with lower inflammation levels and a reduced risk of chronic physical illness [59], while depressive and anxiety symptoms are also linked to physical frailty [60]. 

Among the many chronic diseases that have reported associations with sarcopenia, neurodegenerative dementia may also be explained in the context of this inflammatory-mediated mechanism. A recent meta-study analyzed 63 journals and found a significantly higher prevalence of sarcopenia in patients with dementia (26.4%, 95% CI: 13.6–44.8%) compared to controls (8.3%, 95% CI: 2.8–21.9%) [3]. Beta-amyloid and tau proteins cause glial cells to release inflammatory mediators, leading to synapse damage and dementia [61]. Systemic inflammation affects muscle mass and strength and has been linked to degenerative changes in brain cells. A cross-sectional study using UK biobank data examined cognitive function in nearly 110,000 people with recurrent depression or bipolar disorder and healthy controls and found that maximal muscle strength was associated with higher cognitive function in both depressed and healthy individuals [10]. Similarly, in a study of 470,000 individuals and 1162 schizophrenia patients, strong links were found between maximum muscle strength, visual memory, reaction time and prospective memory in both groups [11]. A study investigating the association between muscle strength and cognitive decline in older Korean adults with normal cognitive function found that muscle strength was positively correlated with MMSE scores and that changes in muscle strength could be used as a predictor of MMSE scores [12]. Considering the close association between cognitive decline and changes in muscle, further research is needed to explore the relationship between LMM and anxiety symptoms, taking into account the cognitive impairment status of the subjects.

The gender differences in this study can be attributed to the effects of sex hormones on skeletal muscle mass. In terms of the role of sex hormones on muscle, estrogen protects skeletal muscle by reducing inflammation, while testosterone increases skeletal muscle strength through anabolism [3,43,44]. Estrogen levels are low in post-traumatic stress disorder [62], while women experiencing anxiety symptoms reported higher frequencies of hot flashes post-menopause [63,64]. Conversely, low testosterone levels are associated with fatigue, low energy and decreased sexual performance. One study found significantly lower salivary testosterone levels in patients with depression and anxiety disorders and increased salivary testosterone levels in patients using SSRIs [65]. High inflammatory levels (hs-CRP) have also been identified in women with LMM and high body fat percentage [66]. Testosterone deficiency is associated with increased pro-inflammatory cytokines, and testosterone supplementation has been shown to reduce anti-inflammatory cytokines (IL-10) and pro-inflammatory cytokines (IL-1β, IL-6, and TNF-α) in patients with coronary artery disease, prostate cancer and diabetes [67]. In other words, in both men and women, increased anxiety is associated with lower levels of estrogen and testosterone, which can be associated with lower muscle mass through mechanisms such as anabolism maintenance of skeletal muscle and increased inflammatory responses. Relating to the findings of this study, the association between LMM and increased anxiety symptoms may be more dominant in men, especially those with more fluctuating testosterone levels.

This study included sociodemographic variables showing differences in the demographic analysis as covariates in the main analysis. The strongest association between LMM and anxiety symptoms was found in Model 3, which additionally adjusted for smoking, alcohol and exercise. Regarding the correlation between smoking and anxiety disorders, as highlighted in prior studies [68], childhood smoking not only escalates the risk of anxiety disorders but also individuals with anxiety disorders tend to use smoking as a means of coping with their anxiety [69,70]. Alcohol use disorders frequently coincide with anxiety disorders [71]. Alcohol intake has been shown to induce dose-dependent myocyte atrophy and death, mediated by alterations in energy metabolism, signal transduction, apoptosis and gene disruption [72]. Finally, physical activity, especially muscle strengthening, is directly related to muscle mass. Both cross-sectional and prospective studies revealed that moderate physical activity reduces the risk of anxiety by 9.3% and 6.3%, respectively, lowering the prevalence and incidence of generalized anxiety disorder by 17%, even after adjusting for confounding variables [73,74]. Previous studies have shown that resistance exercise training (RET) positively improves muscle strength, reducing anxiety symptoms in older adults [39,75]. In younger adults, RET has been reported to have anti-anxiety effects [76]. Adolescents with higher levels of strength have been shown to have lower rates of psychiatric diagnoses and a lower risk of death by suicide [77], suggesting that exercise not only has physiological benefits but also has psychological effects, including increased adaptability to stressful situations, self-transcendence and social integration [78]. Consistent with these findings, our analysis in this study may have been influenced by the substantial impact of smoking, alcohol and exercise factors on anxiety disorders. Further studies are required to analyze the contributions of these factors to the main findings.

There are some chronic diseases and conditions with which LMM, as well as anxiety disorders, can be associated. Low skeletal muscle mass (LMM) is known to be associated with negative outcomes in cardiovascular diseases [3,6], stroke [8] and cancer [4,6,7], which are also demonstrated to be prevalent among individuals exhibiting anxiety symptoms by the large systematic literature [79]. In this study, participants with possible pathologies or diseases such as cardiovascular diseases, stroke or malignancy were excluded. In addition, following previous research, we adjusted socio-demographic factors (age, sex); lifestyle factors (smoking status, heavy alcohol consumption, regular physical exercise); and health-related factors (hypertension, fasting plasma glucose) [34,80]. Therefore, we assume that the participants in this study are apparently healthy adults and have adjusted relevant variables. This study has the following strengths. First, multicenter health examination data were used to obtain a relatively large sample size (*n* = 174,262), allowing for the generation of statistically significant results. Second, while previous studies have primarily assessed sarcopenia in older adults, this study analyzed a younger population, allowing for correlations across all age groups (mean age: 41.7 ± 9.4). Third, unlike previous studies, this study conducted gender-specific analyses to examine the association between LMM and anxiety and attempted to delineate further differences based on gender characteristics. Fourth, unlike previous studies that mainly analyzed the association of anxiety symptoms with muscle strength during sarcopenia, this study has the advantage of assessing skeletal muscle mass to determine the mechanism of direct muscle mass loss. Fifth, this study corrected for confounding variables known to be associated with LMM and attempted to delineate further the effects of smoking, alcohol and exercise variables on anxiety symptoms and muscle mass loss. Sixth, this study excluded participants with a history of chronic diseases such as malignancy, cardiovascular disease, tuberculosis, chronic obstructive pulmonary disease and cirrhosis, allowing for analysis within a healthy population to exclude the effects of chronic disease on muscle mass loss and anxiety.

Nevertheless, this study has several limitations, as follows. First, this is a retrospective and cross-sectional study, which has the fundamental limitation of being unable to test for causality. Further longitudinal studies are needed to investigate the causal relationship between LMM and anxiety symptoms. Second, because this study utilized health examination data, the sample was largely middle-aged Korean men and women, which may have led to a selection-bias. However, the age of the population showed a normal distribution in the bell curve. Moreover, we performed multivariate-adjusted analysis including age as a possible confounding factor. Third, this study utilizes data collected over a relatively short period (two years, 2018–2019). It might have led to a selection bias because it might have missed all the cases who have not attend the center during this period. However, as a two-center study in a large-scale sample, a distinct association between LMM and anxiety symptoms was confirmed. LMM and anxiety symptoms are chronic condition. Fourth, the dataset did not contain data on the presence of anxiety disorders, which means individuals with underlying anxiety disorders who were not taking any anxiolytics may have been included. This could have impacted the study results, and the cohort may not have consisted entirely of healthy individuals. It is necessary to interpret the results of this study with caution and obtain diagnostic history data on anxiety disorders for future research. Fifth, this study used only one method of estimating skeletal muscle mass, using bioimpedance, even though many other methods, such as appendicular muscle mass divided by height, weight, BMI or total muscle mass divided by body weight. Further comparative analyses of different methods of estimating skeletal muscle mass are needed to confirm the clear association between muscle mass and anxiety symptoms. Finally, our utilization of self-reported anxiety assessments might have introduced response bias, despite our efforts to exclude careless responses. Future investigations could improve precision by employing objective measures to examine the influence of LMM on the prevalence of anxiety symptoms.

## 5. Conclusions

The study found that in healthy men and women, LMM was significantly associated with increased anxiety symptoms, with stronger association observed in men than women. Further longitudinal research with enhanced data precision is necessary to determine the causality between LMM and anxiety symptoms. It is essential to obtain diagnostic history data on anxiety disorders and validate the predictive effectiveness of LMM in relation to anxiety disorders, gaining a comprehensive understanding of the underlying pathophysiological mechanisms for future research.

## Figures and Tables

**Table 1:** Baseline characteristics of study participants.

Characteristics	Overall	Female	Male	*p*-Value
Number	174,262	73,833 (42.37)	100,429 (57.63)	
Age (year)	42.25 ± 8.48	41.57 ± 8.42	42.75 ± 8.5	<0.001 ***
Screening center (Seoul, %)	59,997 (34.43)	23,732 (32.14)	36,265 (36.11)	<0.001 ***
Height (cm)	168.11 ± 8.41	160.73 ± 5.22	173.54 ± 5.77	<0.001 ***
Weight (kg)	67.73 ± 13.52	57.17 ± 8.96	75.5 ± 10.77	<0.001 ***
BMI (kg/m^2^)	23.81 ± 3.49	22.13 ± 3.29	25.04 ± 3.1	<0.001 ***
Appendicular Skeletal Muscle Mass (kg)	20.9 ± 4.92	16.05 ± 2.11	24.47 ± 2.94	<0.001 ***
SMI (kg/m^2^) *	7.29 ± 1.14	6.2 ± 0.61	8.1 ± 0.65	<0.001 ***
Smoker status				<0.001 ***
Never smoker	89,666 (51.45)	65,624 (88.88)	24,042 (23.94)	
Former smoker	57,437 (32.96)	6257 (8.47)	51,180 (50.96)	
Current smoker	25,993 (14.92)	1046 (1.42)	24,947 (24.84)	
Heavy alcohol	50,798 (29.15)	7552 (10.23)	43,246 (43.06)	<0.001 ***
Regular physical exercise	25,764 (14.78)	9310 (12.61)	16,454 (16.38)	<0.001 ***
Comorbidities				
Diabetes (%)	5813 (3.34)	1186 (1.61)	4627 (4.61)	<0.001 ***
Hypertension (%)	18,007 (10.33)	3220 (4.36)	14,787 (14.72)	<0.001 ***
Dyslipidemia (%)	30,063 (17.25)	6628 (8.98)	23,435 (23.33)	<0.001 ***
Laboratory Finding				
Fasting plasma glucose (mg/dL)	98.13 ± 15.41	94.37 ± 12.71	100.9 ± 16.59	<0.001 ***
HbA1c (%)	5.51 ± 0.55	5.41 ± 0.46	5.59 ± 0.61	<0.001 ***
Fasting insulin (µU/mL)	7.72 ± 5.01	7.07 ± 4.7	8.19 ± 5.18	<0.001 ***
Total Cholesterol (mg/dL)	190.03 ± 33.97	185.64 ± 32.49	193.26 ± 34.67	<0.001 ***
LDL-C (mg/dL)	128.39 ± 33.08	120.69 ± 31.56	134.04 ± 33.04	<0.001 ***
HDL-C (mg/dL)	60.31 ± 16.3	68.48 ± 16.17	54.3 ± 13.55	<0.001 ***
Triglycerides (mg/dL)	120.77 ± 83.87	90.48 ± 53.82	143.03 ± 94.37	<0.001 ***
HOMA-Ir ^†^	1.92 ± 1.47	1.7 ± 1.32	2.09 ± 1.55	<0.001 ***
Systolic blood pressure (mmHg)	110.68 ± 12.63	104.81 ± 11.73	115 ± 11.47	<0.001 ***
Diastolic blood pressure(mmHg)	71.25 ± 9.76	66.77 ± 8.74	74.54 ± 9.15	<0.001 ***

Data are presented as mean ± standard deviation, number (percentage), or median (interquartile range). *p* values for the between-group difference by *t*-test and Mann–Whitney U test in continuous variables or by Chi-square test in categorical variables. *** *p* < 0.001. BMI = body mass index, SMI = skeletal muscle mass index, * SMI (kg/m^2^) = appendicular skeletal muscle mass (kg)/height (m^2^), HbA1c = glycated hemoglobin, LDL-C = high-density lipoprotein cholesterol, HDL-C = high-density lipoprotein cholesterol, HOMA-Ir = Homeostatic Model Assessment for Insulin Resistance, ^†^ HOMA-Ir = fasting insulin (µU/mL) × fasting plasma glucose (mg/dL).

**Table 2:** Baseline characteristics of study participants classified by the presence of Low skeletal muscle mass.

Characteristics	Female		Effect Size (95% CI)	*p*-Value	Male		Effect Size (95% CI)	*p*-Value
	Control	LMM			Control	LMM		
Number	58,942 (79.83)	14,891 (20.17)			96,551 (96.14)	3878 (3.86)		
Age (year)	41.91 ± 8.3	40.25 ± 8.75	0.2 (0.18–0.22)	<0.001	42.67 ± 8.37	44.75 ± 11	−0.24 (−0.28–−0.21)	<0.001 ***
Screening center (Seoul, %)	19,307 (32.76)	4425 (29.72)	0.07 (0.05–0.08)	<0.001	34,966 (36.22)	1299 (33.5)	0.06 (0.02–0.09)	0.001
Height (cm)	161.29 ± 5.19	158.48 ± 4.72	0.55 (0.53–0.57)	<0.001	173.72 ± 5.71	169 ± 5.43	0.83 (0.8–0.86)	<0.001 ***
Weight (kg)	59.28 ± 8.61	48.81 ± 4.16	1.32 (1.3–1.34)	<0.001	76.18 ± 10.37	58.58 ± 5.32	0.83 (0.8–0.86)	<0.001 ***
BMI (kg/m^2^)	22.8 ± 3.25	19.46 ± 1.72	1.11 (1.1–1.13)	<0.001	25.22 ± 3	20.54 ± 1.91	1.58 (1.54–1.61)	<0.001 ***
Appendicular Skeletal Muscle Mass (kg)	16.66 ± 1.84	13.63 ± 1.09	1.76 (1.74–1.78)	<0.001	24.68 ± 2.78	19.28 ± 1.52	1.97 (1.93–2)	<0.001 ***
SMI (kg/m^2^) *	6.39 ± 0.51	5.42 ± 0.24	2.08 (2.06–2.1)	<0.001	8.16 ± 0.6	6.74 ± 0.25	2.39 (2.36–2.42)	<0.001 ***
Smoker status				<0.001				<0.001 ***
Never smoker	52,181 (88.53)	13,443 (90.28)			22,948 (23.77)	1094 (28.21)		
Former smoker	5161 (8.76)	1096 (7.36)			49,374 (51.14)	1806 (46.57)		
Current smoker	855 (1.45)	191 (1.28)	0.01 (0–0.03)		23,993 (24.85)	954 (24.6)	0.01 (−0.03–0.04)	
Heavy alcohol	6130 (10.4)	1422 (9.55)	0.03 (0.01–0.05)	<0.001	41,862 (43.36)	1384 (35.69)	0.15 (0.12–0.19)	<0.001 ***
Regular Physical exercise	8043 (13.65)	1267 (8.51)	0.16 (0.14–0.17)	<0.001	16,025 (16.6)	429 (11.06)	0.15 (0.12–0.18)	<0.001 ***
Comorbidities								
Diabetes (%)	991 (1.68)	195 (1.31)	0.03 (0.01–0.05)	0.001	4411 (4.57)	216 (5.57)	−0.05 (−0.08–−0.02)	0.004
Hypertension (%)	2771 (4.7)	449 (3.02)	0.08 (0.06–0.1)	<0.001	14,353 (14.87)	434 (11.19)	0.1 (0.07–0.14)	<0.001 ***
Dyslipidemia (%)	5423 (9.2)	1205 (8.09)	0.04 (0.02–0.06)	<0.001	22,693 (23.5)	742 (19.13)	0.1 (0.07–0.14)	<0.001 ***
Laboratory Finding								
Fasting glucose (mg/dL)	94.88 ± 13.14	92.36 ± 10.63	0.2 (0.18–0.22)	<0.001	100.98 ± 16.46	98.95 ± 19.48	0.12 (0.09–0.15)	<0.001 ***
HbA1c (%)	5.43 ± 0.47	5.33 ± 0.38	0.21 (0.19–0.23)	<0.001	5.59 ± 0.6	5.55 ± 0.72	0.07 (0.03–0.1)	<0.001 ***
Fasting insulin (µU/mL)	7.37 ± 5.01	5.89 ± 2.92	0.32 (0.3–0.34)	<0.001	8.29 ± 5.22	5.66 ± 3.14	0.51 (0.48–0.54)	<0.001 ***
Total Cholesterol (mg/dL)	185.62 ± 32.51	185.7 ± 32.39	0 (−0.02–0.02)	0.79	193.39 ± 34.67	189.91 ± 34.57	0.1 (0.07–0.13)	<0.001 ***
LDL-C (mg/dL)	121.23 ± 31.68	118.53 ± 31.01	0.09 (0.07–0.1)	<0.001	134.25 ± 32.98	128.79 ± 34	0.17 (0.13–0.2)	<0.001 ***
HDL-C (mg/dL)	67.45 ± 16.13	72.56 ± 15.67	−0.32 (−0.34–−0.3)	<0.001	54.05 ± 13.41	60.74 ± 15.25	−0.5 (−0.53–−0.46)	<0.001 ***
Triglycerides (mg/dL)	92.94 ± 56.61	80.75 ± 39.51	0.23 (0.21–0.25)	<0.001	144.08 ± 94.88	116.93 ± 75.98	0.29 (0.26–0.32)	<0.001 ***
HOMA-Ir ^†^	1.78 ± 1.41	1.37 ± 0.76	0.32 (0.3–0.33)	<0.001	2.12 ± 1.56	1.41 ± 0.92	0.46 (0.43–0.49)	<0.001 ***
Systolic blood pressure (mmHg)	105.76 ± 11.86	101.08 ± 10.41	0.4 (0.39–0.42)	<0.001	115.22 ± 11.42	109.46 ± 11.14	0.5 (0.47–0.54)	<0.001 ***
Diastolic blood pressure (mmHg)	67.2 ± 8.85	65.08 ± 8.07	0.24 (0.23–0.26)	<0.001	74.63 ± 9.15	72.21 ± 8.83	0.27 (0.23–0.3)	<0.001 ***

*p* values for the between-group difference by *t*-test and Mann–Whitney U test in continuous variables or by Chi-square test in categorical variables. *** *p* < 0.001. BMI = body mass index, SMI = skeletal muscle mass index, * SMI (kg/m^2^) = appendicular skeletal muscle mass (kg)/height (m^2^), HbA1c = glycated hemoglobin, LDL-C = high-density lipoprotein cholesterol, HDL-C = high-density lipoprotein cholesterol, HOMA-Ir = Homeostatic Model Assessment for Insulin Resistance, ^†^ HOMA-Ir = fasting insulin (µU/mL) × fasting plasma glucose (mg/dL).

**Table 3:** Multivariate regression analyses showing associations of low skeletal muscle mass with the presence of elevated anxiety symptoms in the study.

	Anxiety Level (CUXOS Score)		
	≤20	>20	
Female			
Unadjusted Model	1 (reference)	1.13 (1.08–1.17)	
Model 1	1 (reference)	1.12 (1.08–1.16)	
Model 2	1 (reference)	1.13 (1.09–1.17)	
Model 3	1 (reference)	1.13 (1.08–1.17)	
Male			
Unadjusted Model	1 (reference)	1.16 (1.07–1.25)	
Model 1	1 (reference)	1.15 (1.06–1.25)	
Model 2	1 (reference)	1.16 (1.07–1.25)	
Model 3	1 (reference)	1.17 (1.08–1.27)	
	**Anxiety Level (CUXOS Score)**		
	**≤20**	**21–30**	**>30**
Female			
Unadjusted Model	1 (reference)	1.09 (1.04–1.15)	1.18 (1.12–1.24)
Model 1	1 (reference)	1.1 (1.05–1.15)	1.15 (1.09–1.22)
Model 2	1 (reference)	1.1 (1.05–1.15)	1.17 (1.1–1.23)
Model 3	1 (reference)	1.1 (1.05–1.15)	1.17 (1.11–1.24)
Male			
Unadjusted Model	1 (reference)	1.1 (1–1.2)	1.29 (1.14–1.46)
Model 1	1 (reference)	1.08 (0.98–1.18)	1.31 (1.16–1.49)
Model 2	1 (reference)	1.08 (0.98–1.19)	1.34 (1.18–1.51)
Model 3	1 (reference)	1.09 (0.99–1.2)	1.35 (1.19–1.53)
	**Anxiety Level (CUXOS Score)**		
	**≤20**	**21–40**	**>40**
Female			
Unadjusted Model	1 (reference)	1.12 (1.07–1.16)	1.2 (1.09–1.32)
Model 1	1 (reference)	1.12 (1.07–1.16)	1.14 (1.04–1.26)
Model 2	1 (reference)	1.12 (1.08–1.17)	1.17 (1.06–1.28)
Model 3	1 (reference)	1.12 (1.08–1.17)	1.18 (1.07–1.3)
Male			
Unadjusted Model	1 (reference)	1.15 (1.06–1.24)	1.27 (0.99–1.63)
Model 1	1 (reference)	1.14 (1.05–1.23)	1.32 (1.03–1.69)
Model 2	1 (reference)	1.14 (1.05–1.24)	1.34 (1.05–1.72)
Model 3	1 (reference)	1.15 (1.06–1.25)	1.36 (1.06–1.74)

CUXOS = Clinically Useful Anxiety Outcome Scale. LMM = low skeletal muscle mass. Odds Ratios (ORs) were calculated as the risks of having mild, moderate and severe anxiety symptoms according to the presence of low skeletal muscle mass (LMM). Model 1: adjusted for age, center. Model 2: Model 1 + history of hypertension, fasting plasma glucose, Model 3: Model 2 + smoking status, heavy alcohol consumption and regular physical exercise. Data are presented as numbers (percentages).

## Data Availability

The data that support the findings of this study are available from the corresponding authors upon reasonable request. The data are not publicly available due to ethical restrictions that protect patient privacy and consent.

## Supplementary Materials

The following supporting information can be downloaded at: https://www.mdpi.com/article/10.3390/brainsci14050438/s1, Table S1: Prevalence of clinical anxiety symptoms in control and low skeletal muscle mass.
