# Supplementary material for: Exploring the Association between Elevated Anxiety Symptoms and Low Skeletal Muscle Mass among Asymptomatic Adults: A Population-Based Study in Republic of Korea
Source: Brain Sci. 2024 Apr 28;14(5):438. doi: 10.3390/brainsci14050438 (PMC11119912; doi:10.3390/brainsci14050438)
Supplement: Supplementary file 1 [file brainsci-14-00438-s001.zip › brainsci-2939758-supplementary.pdf]

**Supplementary Table S1**

Prevalence of clinical anxiety symptoms in control and low skeletal muscle mass.

| | Total | Anxiety level (CUXOS score) | | *p*-value |
|---|---|---|---|---|
| | | ≤20 | >20 | |
| Total | | | | <0.001*** |
| Control | 155493 (89.23) | 119510 (76.86) | 35983 (23.14) | |
| LMM | 18769 (10.77) | 13072 (69.65) | 5697 (30.35) | |
| Female | | | | <0.001*** |
| Control | 58942 (79.83) | 41159 (69.83) | 17783 (30.17) | |
| LMM | 14891 (20.17) | 10015 (67.26) | 4876 (32.74) | |
| Male | | | | <0.001*** |
| Control | 96551 (96.14) | 78351 (81.15) | 18200 (18.85) | |
| LMM | 3878 (3.86) | 3057 (78.83) | 821 (21.17) | |

| | Anxiety level (CUXOS score) | | | p-value |
|---|---|---|---|---|
| | ≤20 | 21-30 | >30 | |
| Total | | | | <0.001*** |
| Control | 119510 (76.86) | 23412 (15.06) | 12571 (8.08) | |
| LMM | 13072 (69.65) | 3427 (18.26) | 2270 (12.09) | |
| Female | | | | <0.001*** |
| Control | 41159 (69.83) | 10858 (18.42) | 6925 (11.75) | |
| LMM | 10015 (67.26) | 2890 (19.41) | 1986 (13.34) | |
| Male | | | | <0.001*** |
| Control | 78351 (81.15) | 12554 (13.00) | 5646 (5.85) | |
| LMM | 3057 (78.83) | 537 (13.85) | 284 (7.32) | |

| | Anxiety level (CUXOS score) | | | *p*-value |
|---|---|---|---|---|
| | ≤20 | 21-40 | >40 | |
| Total | | | | <0.001*** |
| Control | 119510 (76.86) | 32595 (20.96) | 3388 (2.18) | |
| LMM | 13072 (69.65) | 5035 (26.83) | 662 (3.53) | |
| Female | | | | <0.001*** |
| Control | 41159 (69.83) | 15748 (26.72) | 2035 (3.45) | |
| LMM | 10015 (67.26) | 4281 (28.75) | 595 (4.00) | |
| Male | | | | 0.001 |
| Control | 78351 (81.15) | 16847 (17.45) | 1353 (1.40) | |
| LMM | 3057 (78.83) | 754 (19.44) | 67 (1.73) | |

Data are presented as numbers (percentages). P values for the between-group difference by Chi-square test. ***P value < 0.001

CUXOS = Clinically Useful Anxiety Outcome Scale, LMM = low skeletal muscle mass.
